# Growth Differentiation Factor-15 (GDF-15) Levels Are Associated with Cardiac and Renal Injury in Patients Undergoing Coronary Artery Bypass Grafting with Cardiopulmonary Bypass

**DOI:** 10.1371/journal.pone.0105759

**Published:** 2014-08-29

**Authors:** Abdelkader Kahli, Charles Guenancia, Marianne Zeller, Sandrine Grosjean, Karim Stamboul, Luc Rochette, Claude Girard, Catherine Vergely

**Affiliations:** 1 Institut National de la Santé et de la Recherche Médicale (Inserm) U866, Laboratoire de Physiopathologie et Pharmacologie Cardio-Métaboliques (LPPCM), Université de Bourgogne, Facultés des Sciences de la Santé, Dijon, France; 2 Service d’Anesthésie-Réanimation, Centre Hospitalier Régional Bocage Central, Dijon, France; 3 Service de Cardiologie, Centre Hospitalier Régional Bocage Central, Dijon, France; University Medical Center Utrecht, Netherlands

## Abstract

**Objective:**

Growth differentiation factor-15 (GDF-15) has been identified as a strong marker of cardiovascular disease; however, no data are available concerning the role of GDF-15 in the occurrence of organ dysfunction during coronary artery bypass grafting (CABG) associated with cardiopulmonary bypass (CPB).

**Methods:**

Five arterial blood samples were taken sequentially in 34 patients from anesthesia induction (IND) until 24 h after arrival at the intensive care unit (ICU). Plasma levels of GDF-15, follistatin-like 1 (FLST1), myeloperoxidases (MPO), hydroperoxides and plasma antioxidant status (PAS) were measured at each time-point. Markers of cardiac (cardiac-troponin I, cTnI) and renal dysfunction (neutrophil gelatinase-associated lipocalin, NGAL) and other classical biological factors and clinical data were measured.

**Results:**

Plasma GDF-15 levels increased gradually during and after surgery, reaching nearly three times the IND levels in the ICU (3,075±284 ng/L vs. 1,061±90 ng/L, p<0.001). Plasma MPO levels increased dramatically during surgery, attaining their highest level after unclamping (UNCLAMP) (49±11 ng/mL vs. 1,679±153 ng/mL, p<0.001) while PAS significantly decreased between IND and UNCLAMP (p<0.05), confirming the high oxidative status induced by this surgical procedure. ICU levels of GDF-15 correlated positively with cTnI and NGAL (p = 0.006 and p = 0.036, respectively), and also with hemoglobin and estimated glomerular filtration rate (eGFR). Among all the post-operative biomarkers available, only eGFR, NGAL and GDF-15 measured at ICU arrival were significantly associated with the onset of acute kidney injury (AKI). Patients with a EuroSCORE >3 were shown to have higher GDF-15 levels.

**Conclusions:**

During cardiac surgery associated with CPB, GDF-15 levels increased substantially and were associated with markers of cardiac injury and renal dysfunction.

## Introduction

During cardiopulmonary bypass (CPB), pro-inflammatory mediators activate leucocytes, vascular endothelial cells and platelets, thus producing a systemic inflammatory response that results in organ dysfunction, affecting the heart, brain, lungs and kidneys. Several factors, including operative trauma, the contact of blood components with the artificial surface of the circuit, cardioplegic techniques and allogenic blood transfusion account for this phenomenon. The cellular response is mediated by the main effectors of the inflammatory response, including neutrophils, which degranulate and release cytotoxic molecules such as elastase, myeloperoxidase (MPO) and free radicals[1]. Several therapeutic strategies have thus been developed to minimize this inflammation and lessen organ dysfunction[2]. In a previous study carried out in our laboratory, we measured systemic and coronary sinus free-radical release during and following removal of the cross-clamp[3]. We observed that radical production was significantly greater during and after the CPB procedure, and that circulating free-radical levels correlated with serum creatine kinase-MB (CK-MB). Various biological and hemodynamic markers are measured to estimate the pre-and postoperative risk of developing complications; however, no data exist to assess the role and impact of growth differentiation factor-15 (GDF-15) in the occurrence of organ dysfunction during coronary artery bypass grafting (CABG) associated with CPB.

GDF-15 (also known as NAG-1 and MIC-1) is a cytokine related to the superfamily of transforming growth factor-β (TGF-β), and is weakly expressed or not expressed at all under physiological conditions[4]. The plasma concentration of GDF-15 increases under pathological conditions such as hypoxia, inflammation or oxidative stress and is closely associated with all-cause mortality[5]. The expression of GDF-15 has been induced in cultured cardiomyocytes during experimental ischemia/reperfusion (IR), and in cardiomyocytes subjected to nitrosative stress and stimulation with proinflammatory cytokines and interferon-γ (IFN-γ)[6]. In the cardiovascular system, GDF-15 is now identified as a strong prognosis marker in patients with cardiovascular disease, including coronary artery disease (CAD), acute coronary syndromes (ACS)[7], [8] and heart failure (HF)[9]. GDF-15 is associated with reduced endothelium-dependent vasodilation, the risk of atherosclerotic plaque rupture and reduced left ventricular ejection fraction (LVEF)[10]. Cardiomyocytes have been identified as the main source of GDF-15 in patients with ST-elevation myocardial infarction (STEMI) or HF[6]. Recent findings also suggested that the secreted protein FSTL-1 could be an upstream inducer of GDF15 production and an independent prognostic biomarker in acute coronary syndromes[11]. Finally, it was suggested that GDF-15 could represent a novel risk marker in association with the EuroSCORE for risk stratification in cardiac surgery patients[12]. In addition, GDF-15 is also a novel independent serum marker of mortality in chronic kidney disease (CKD), and combining this marker with other established predictors of mortality may help to identify individuals at high risk for developing CKD [13], [14].

The present study was designed to evaluate the kinetics of plasma GDF-15 levels in patients with significant CAD undergoing cardiac surgery associated with CPB, and to relate GDF-15 to inflammatory/ oxidative stress status and to organ dysfunction.

## Methods

### Study patients

Thirty-four consecutive patients operated on for CABG under CPB at Dijon University Hospital, Bocage Central, Dijon, France were included in this prospective study between April 26^th^ 2012 and October 11^th^ 2012. The study protocol complied with the Declaration of Helsinki and was approved by the regional ethics committee (Espace éthique de Bourgogne/Franche-Comté, CHU de Besançon, Hôpital St Jacques, Besançon, France; ARTICLE Protocol, 2012-A00184-39). Informed written consent was obtained from all of the patients. Patients with stable CAD were included. The following criteria led to the exclusion of patients: surgical emergencies, aortic valve replacement, ACS reported within 30 days before the surgery, LVEF <30%, chronic inflammatory pathologies, infectious or malignant diseases, chronic renal failure, transplant patients and patients treated with corticosteroids.

### Clinical postoperative events

Clinical events were defined as hospital stay>10 days, intensive care unit (ICU) stay>5 days, the use of vasopressor therapy and clinical complications (blood transfusion, infection, acute kidney injury and atrial fibrillation).

The association between these events and biomarkers was also analyzed.

### Anesthesia and heart surgery procedure

Patients were pre-medicated with midazolam orally plus hydroxyzine 90 min before anesthesia. Routine cardiac medications were continued until the morning of the surgery, except for Clopidogrel, which was stopped at least 5 days earlier. Before the induction of anesthesia, a complete hemodynamic monitoring system was set up in the operating room. Anesthesia was induced with intravenous midazolam (0.02 mg.kg^−1^), sufentanil (0.2 to 0.5 µg.kg^−1^.h^−1^), and propofol (1.5 to 2.5 mg.kg^−1^). After verifying that manual ventilation was satisfactory, cisatracurium dibesylate (0.06 mg.kg^−1^.h^−1^) was injected. Patients were orally intubated and ventilated with FiO_2_: 0.4. Anesthesia was maintained with sufentanil and cisatracurium as required and inhaled desflurane.

Surgery was performed according to the following protocol: sternotomy, harvesting of the right and left internal mammary arteries and saphenous vein, and aortic and cavoatrial cannulation, followed by the implementation of CPB and myocardial protection by anterograde cardioplegia. Moderate body hypothermia (32°C) was used. The time from removal of the aortic cross-clamp until the discontinuation of CPB was measured. After completion of the CABG (from 1 to 5 CABG in the patients studied), CPB was discontinued and protamine was given for heparin reversal. Patients were given a blood transfusion in the case of severe anemia (blood hematocrit, 22% during CPB or, 26% after CPB). After closure of the sternum, the patients were transferred to the post-operative ICU, and finally to the surgery ward. Clinical and biological parameters were regularly recorded. Complications such as arrhythmia, blood loss, infection, and organ failure were recorded. ICU length of stay was noted.

### Study protocol

**Figure 1:**
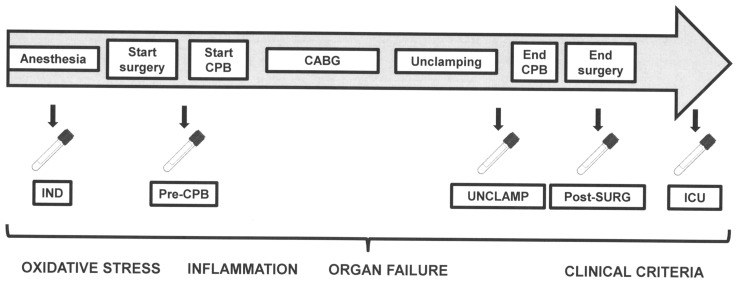
Schematic diagram of the study protocol. Arterial blood samples were harvested after anesthesia (IND), just before starting CPB (pre-CPB), just after removing the cross clamp (UNCLAMP), after surgery upon arrival in the ICU (Post-SURG) and after 24 hours at the cardiovascular intensive care unit (ICU). Markers of oxidative stress, inflammation, organ failure and clinical criteria were collected.

Blood samples were taken in heparinized tubes as shown in Figure 1. Arterial samples were taken from the arterial catheter or from the CPB pump: after anesthesia (IND), just before starting CPB (pre-CPB), just after removing the cross clamp (UNCLAMP), after surgery (Post-SURG) upon arrival and after 24 hours at the ICU. Blood samples were immediately centrifuged after collection and the plasma was immediately frozen in liquid nitrogen and stored at −80°C until analysis.

### Plasma levels of GDF-15 and follistatin like-1 during cardiac surgery with CPB

Plasma GDF-15 concentrations were measured by quantitative sandwich enzyme immunoassay (Human GDF-15, Quantikine, R&D Systems Europe, Lille, France) with a linear range from 200 to 50,000 ng/L. The color intensity, relative to GDF-15 concentration, was measured at 450 nm with a spectrophotometer (VictorV3, Perkin Elmer, Courtaboeuf, France). A GDF-15 value of 1,200 ng/L corresponds to the upper limit of normal in healthy elderly individuals, whereas GDF-15 levels of 1,200 and 1,800 ng/L allowed the identification of patients at low (<1,200 ng/L), intermediate (between 1,200 and 1,800 ng/L), or high risk (>1,800 ng/L)[7], [15]. Plasma follistatin like-1 protein (FSTL-1) concentrations were measured by a quantitative sandwich enzyme immunoassay technique (ELISA kit for FSTL-1, Uscn, Life Science Inc). In order to account for hemodilution, plasma GDF-15 and FSTL-1 levels were corrected using proteinemia.

### Oxidative stress/inflammation analysis

The plasma antioxidant status (PAS) was determined by measuring the Oxygen Radical Absorbance Capacity (ORAC), as detailed elsewhere[3], [16]. The results were expressed in ORAC units, where 1 ORAC unit equals the net protection provided by 1 µM Trolox. Plasma antioxidant status is a dynamic measurement and does not need to be corrected for hemodilution. Plasma MPO concentrations were measured by a quantitative sandwich enzyme immunoassay technique (Human Myeloperoxidase Immunoassay, Quantikine ELISA, R&D Systems Europe, Lille, France). Plasma concentrations of hydroperoxides were determined by a colorimetric assay using Free Oxygen Radical Testing (FORT, FORM-PLUS-3000, Optimabio, Ollioules, France), as previously described[17]. The hydroperoxide concentrations were corrected for hemodilution.

Plasma protein concentrations (expressed as grams per liter of plasma) were evaluated by the spectrophotometric method according to Lowry *et al*.

### Markers of organ dysfunction

Neutrophil gelatinase-associated lipocalin (NGAL) is considered an early marker of acute kidney injury (AKI)[18], [19]. Plasma NGAL concentrations were measured at IND, post-SURG and ICU by a quantitative sandwich enzyme immunoassay technique (Human NGAL Immunoassay, Quantikine ELISA, R&D Systems Europe, Lille, France). Standard blood count, platelet count, renal enzymes, blood gases, pH, lactic acid, cardiac troponin-I (cTnI) were measured by the hospital’s medical analysis laboratory the day before (DAY-1), before (PRE-CPB) and after CPB (post-SURG) and at the first postoperative day (ICU). The following patients’ data were collected: age, sex, and cardiovascular risk factors (hypertension, atrial fibrillation, body mass index (BMI), dyslipidemia, diabetes mellitus and renal failure), current smoker and chronic medications before admission. Hemodynamic parameters on admission (heart rate, systolic blood pressure) were recorded. LVEF was measured by echocardiography at admission and during the postoperative hospital stay. The estimated glomerular filtration rate (eGFR) was calculated from pre-operative plasma creatinine by the abbreviated Modifications of Diet in Renal Disease equation[20]. AKI was defined in accordance with the AKI network (AKIN) criteria as an abrupt decrease (in 48 h) in renal function, defined by an increase in absolute serum creatinine (SCr) of at least 26.5 µmol/L (0.3 mg/dL) or by a percentage increase in SCr ≥50% (1.5× baseline value), or by a decrease in the urinary output (documented oliguria <0.5 mL/kg/h for more than 6 h)[21]. The additive EuroSCORE was calculated, and a cut-off at 3 was used for more clinical relevance.

### Statistical analyses

Continuous variables are presented as mean ± SEM or median (IQR) as appropriate, and discrete variables as absolute number and percentages.

Given time points were analyzed by Student’s un-paired t-test or the Mann-Whitney test for normally or not normally distributed data, respectively. Results are expressed as box plots in which the boxes represent the 25^th^ and 75^th^ percentiles, or as means+SEM.

One Way Repeated Measures Analysis of Variance or Friedman Repeated Measures Analysis of Variance on Ranks was used to analyze differences within groups over time, and corrected for multiple comparisons by the Tukey test. A probability value of <0.05 was considered statistically significant.

Associations between laboratory parameters and GDF-15 were tested using Spearman’s correlation rank test.

To determine the incremental diagnostic value of post-operative GDF-15 in the early detection of AKI compared with conventional biomarkers, a stepwise analysis was performed given the limited number of events. We calculated the improvement in global χ2 after the addition of GDF-15 to a model that included post-operative eGFR and eGFR plus NGAL.

A probability value <0.05 was considered statistically significant.

Statistical analyses were performed using SigmaPlot version 12 (Systat Software, Inc.) and SPSS 20.0 0 (SPSS, Inc., Chicago, IL, USA).

## Results

Perioperative clinical and laboratory information is shown in Table 1 (left part). The mean age of patients was 65±2 years. Preoperative LVEF was 58±2% and the mean EuroSCORE was 2.9. Initial plasma GDF-15 levels (IND) ranged from 456 to 2,832 ng/L with a mean value of 1,061±91 ng/L.

**Table 1:** Association between induction plasma GDF-15 concentration and baseline clinical and biological variables^1^.

		Mean (IQR) of plasma GDF-15 concentration (ng/L)	p	r
Gender	M n = 27 (79%)	961 (605–1401)	0.418	
	F n = 7 (21%)	827 (752–935)		
Age (year)(mean ± SEM)	65.2±1.7		0.005	0.477
Medical history				
Hypertension	Yes n = 25 (74%)	935 (787–1443)	0.101	
	No n = 9 (26%)	761 (572–1092)		
Diabetes	Yes n = 14 (41%)	1002 (881–1672)	0.011	
	No n = 20 (59%)	825 (593–1109)		
Hyperlipidemia	Yes n = 31 (91%)	883 (743–1347)	0.903	
	No n = 3 (9%)	1290 (587–1334)		
Obesity	Yes n = 10 (29%)	935 (853–1745)	0.234	
	No n = 24 (71%)	855 (614–1323)		
Weight (kg)(mean ± SEM)	79.4±2.8		0.109	0.279
BMI (kg/m^2^)(mean ± SEM)	28.0±0.8		0.196	0.226
Smoking	Yes n = 9 (26%)	861 (683–1251)	0.725	
	No n = 25 (74%)	935 (692–1340)		
Chronic renal failure (GFR <60 mL/min/1,73 m^2^)	Yes n = 5 (15%)	1645 (884–2594)	0.046	
	No n = 29 (85%)	877 (674–1174)		
Creatinine (μmol/L)(mean ± SEM)	87.6±3.2		0.019	0.398
Glomerular filtration rate (GFR)	80.4±3.3		0.006	−0.465
(mL/min/1,73 m^2^)(mean ± SEM)				
Urea (mmol/L)(mean ± SEM)	6.9±0.4		0.105	0.282
NGAL (μg/L)(mean ± SEM)	108.3±6.1		0.220	0.215
Infarction	Yes n = 6 (18%)	923 (589–1168)	0.635	
	No n = 28 (82%)	884 (745–1388)		
PCI	Yes n = 6 (18%)	951 (765–1204)	0.874	
	No n = 28 (82%)	881 (667–1344)		
LVEF (%)(mean ± SEM)	58.0±1.9		0.328	−0.172
EuroSCORE(mean ± SEM)	2.9±0.4		0.0003	0.585
Cardiac status				
Troponin>0.02 µg/L	Yes n = 7 (21%)	987 (587–1402)	0.898	
	No n = 27 (79%)	884 (743–1334)		
Creatine kinase (IU/L)(mean ± SEM)	139.7±14.2		0.717	0.064
NT-proBNP (pg/mL)(mean ± SEM)	594.1±113.3		0.044	0.347
Inflammatory and oxidative stress (mean ± SEM)				
CRP>3 mg/L	Yes n = 5 (15%)	1541 (881–2594)	0.041	
	No n = 29 (85%)	861 (623–1208)		
Fibrinogen (g/L)	3.5±0.1		0.100	0.571
Hydroperoxides (mmol/L)	2.1±0.1		0.547	0.106
PAS (ORAC)	3619.2±137.5		0.900	−0.022
Myeloperoxidase (ng/mL)	49.3±10.8		0.216	0.217
Lactate (mmol/L)	1.1±0.1		0.003	−0.490
Biological variables(mean ± SEM)				
Hemoglobin pre op (g/dL)	13.0±0.3		0.156	−0.248
Hematocrit (%)	39.9±0.9		0.137	−0.260
Blood glucose (mmol/L)	7.5±0.8		0.006	0.464
ASAT (IU/L)	22.4±1.2		0.055	−0.332
ALAT (IU/L)	36.7±2.1		0.018	−0.404

1 p-value and Spearman's rank correlation was for used for testing the association of GDF-15 at IND to the baseline clinical and biological variables.

Intraoperative data and postoperative outcomes are shown in Table 2. The majority of patients (n = 21) underwent three bypass grafts. Nine patients with LVEF<40% received an infusion of Glucose-Insulin–Potassium (GIK) (potassium 40 mEq and regular insulin 300 IU in 500 ml of 30% glucose) one hour before aortic clamping. All of the patients were still alive on 31^st^ October 2013.

**Table 2:** Intraoperative data and postoperative outcomes.

No. of CABG, n (%)	
Two	7 (20%)
Three	21 (62%)
Four	4 (12%)
Five	2 (6%)
GIK (Yes/No)	9/25
Cross-clamping time (min)	61.7±3.7
Cardioplegia (min)	52.5±3.6
Defibrillation	
Spontaneous	32 (94%)
Electric	2 (6%)
Postoperative LVEF (%)(mean ± SEM)	57.4±1.2
Number of surgeries with CPB > 120 min, n (%)	2 (6%)
In-hospital stay after surgery (days) (mean ± SEM)	10.3±0.4
Postoperative in-hospital > 10 days, n (%)	10 (29%)
ICU stay (days) (mean ± SEM)	3.7±0.2
ICU stay > 5 days, n (%)	4 (12%)
Clinical complication, n (%)	
Blood transfusion	9 (26%)
Atrial fibrillation (AC/FA)	7 (21%)
AKI	9 (26%)
Use of vasopressor	9 (26%)
Postoperative infection	4 (12%)
SIRS	14 (41%)

### Plasma levels of GDF-15 and FSTL-1 during cardiac surgery with CPB

**Figure 2:**
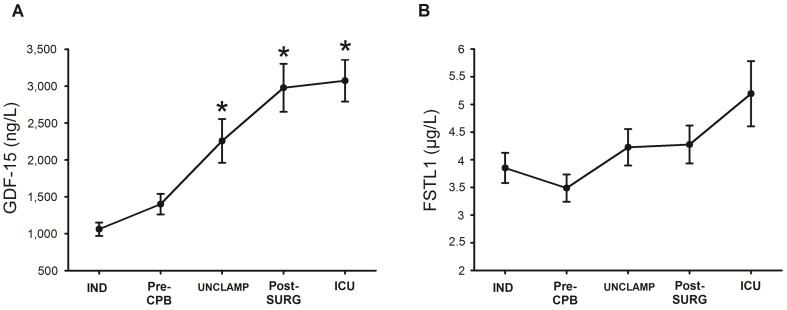
Time course of plasma GDF-15 (A) and FSTL-1 (B) levels in patients (n = 34) undergoing cardiac surgery associated with CPB. Values, corrected for hemodilution, are mean ± SEM, *: significantly different (p<0.05) between time-point and IND levels (One way repeated measures ANOVA, followed by pair-wise comparison of mean levels with Tukey test).

Compared with IND levels, plasma GDF-15 concentrations rose significantly (p<0.05) after removal of the cross-clamp and during the post-surgery period in the ICU, reaching nearly three times the induction level (Figure 2A). Plasma FSTL-1 levels were not modified during the surgical procedure. However, a slight rise in FSTL-1 was observed in the ICU (Figure 2B).

Clinical and biological variables associated with IND plasma GDF-15 concentrations are shown in Table 1 (right part). GDF-15 levels were significantly associated with age (r = 0.477, p = 0.005). A medical history of diabetes mellitus was significantly associated with higher GDF-15 levels (p = 0.011). Moreover, associations between GDF-15 concentrations and blood glucose levels were significant (r = 0.464, p = 0.006). Concerning the cardiac status, plasma levels of GDF-15 were significantly associated with those of N-terminal pro-brain natriuretic peptide (NT-proBNP) (r =  0.347, p = 0.044) but not with plasma levels of creatine kinase.

Although lower GFR values and higher plasma creatinine levels were associated with higher GDF-15 levels (respectively, r = −0.465, p = 0.006 and r = 0.398, p = 0.019), neither urea nor NGAL concentrations were significantly associated with GDF-15 levels. Regarding the inflammatory and oxidative stress status, the highest GDF-15 levels were associated with CRP (p = 0.041), but not with higher hydroperoxide and MPO concentrations or PAS. Higher preoperative GDF-15 levels were significantly associated with higher EuroSCORE values (r = 0.585, p = 0.0003).

**Figure 3:**
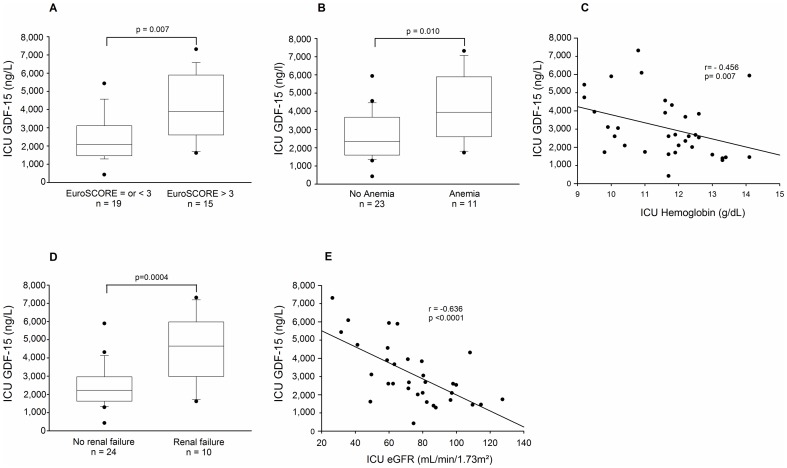
Association of post-operative (ICU) GDF-15 levels with clinical and biological variables: GDF-15 levels in patients (n =  34) according to EuroSCORE ≤ 3 or EuroSCORE > 3(A), GDF-15 levels in patients presenting or not anemia (B), correlation between GDF-15 and plasma hemoglobin levels (C), GDF-15 levels in patients presenting or not renal failure (D), correlation between plasma GDF-15 levels and glomerular filtration rate (E). (Student’s test, Spearman’s correlation).

The association of clinical and biological variables with peak plasma GDF-15 concentrations, observed one day after surgery in ICU, was also investigated. Patients with moderate or high operative risk (EuroSCORE > 3) had higher plasma GDF-15 concentrations than did patients with low operative risk (EuroSCORE ≤3) (p = 0.007) (Figure 3A). GDF-15 levels at the ICU were significantly higher in patients who presented anemia during the postoperative period (p = 0.010) (Figure 3B); moreover, higher GDF-15 concentrations correlated with lower ICU plasma hemoglobin (r = −0.456, p = 0.007) (Figure 3C). Patients who developed postoperative renal failure presented significantly higher GDF-15 levels at the ICU (p = 0.0004) (Figure 3D). Furthermore, ICU levels of GDF-15 correlated significantly with a worse eGFR (r = −0.640, p<0.0001) (Figure 3E).

### Evolution of oxidative stress/inflammation

**Figure 4:**
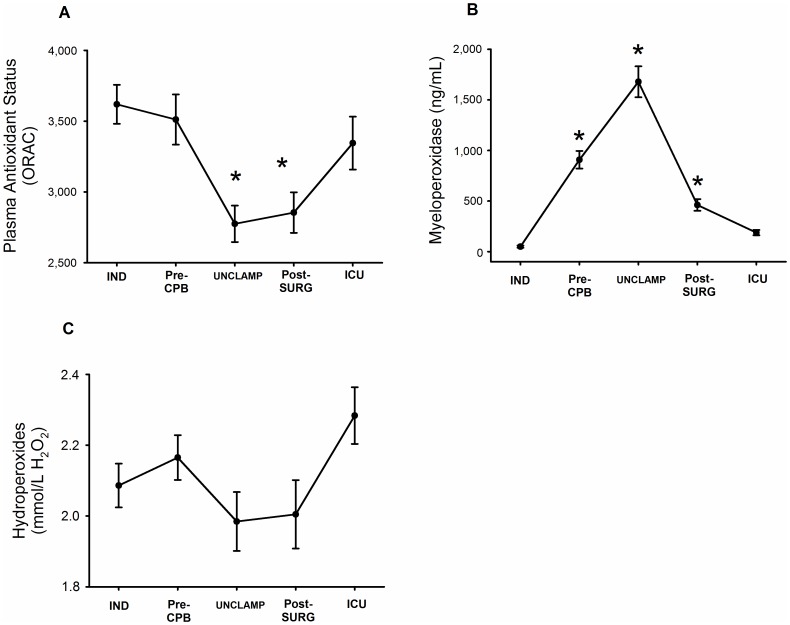
Time course of plasma antioxidant status (A), myeloperoxidase (B) and hydroperoxides (C) given as the mean ± SEM, n = 34, *: significantly different (p<0.05) between time-point and IND levels (One way repeated measures ANOVA followed by pair-wise comparison of mean levels with Tukey test).

The PAS decreased significantly (p<0.05) during the CPB procedure (Figure 4A) while plasma MPO levels increased dramatically (p<0.05) during the surgical procedure, attaining their highest levels just after unclamping and then decreasing rapidly (Figure 4B), confirming the high inflammatory status induced by this surgical procedure. Whereas plasma hydroperoxide levels did not fluctuate significantly during and after the surgery (Figure 4C), ICU hydroperoxide plasma levels were associated with longer ICU and hospital stays (p = 0.027 and p = 0.044 respectively).

### GDF-15 and Markers of tissue injury

**Figure 5:**
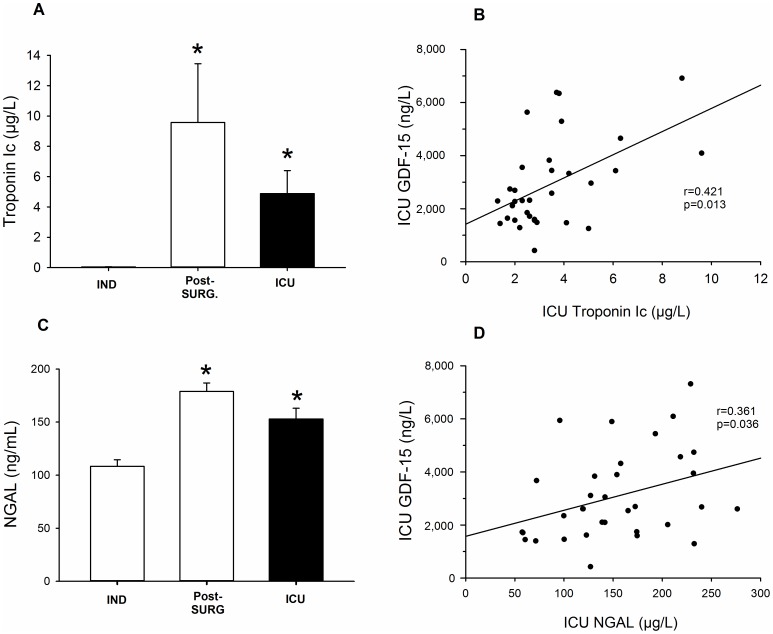
Correlations between post-operative ICU GDF-15 levels and markers of renal and cardiac injury: plasma levels of cTnI (μg/L) at IND, post-SURG and ICU (A), correlation between plasma levels of GDF-15 and cTnI ICU (B), plasma levels of NGAL (ng/mL) at IND, post-SURG and ICU (C), correlation between plasma levels of GDF-15 and NGAL at the ICU (D). (One way repeated measures ANOVA followed by pair-wise comparison of mean levels with Tukey test; Spearman’s correlation, n = 34).

**Figure 6:**
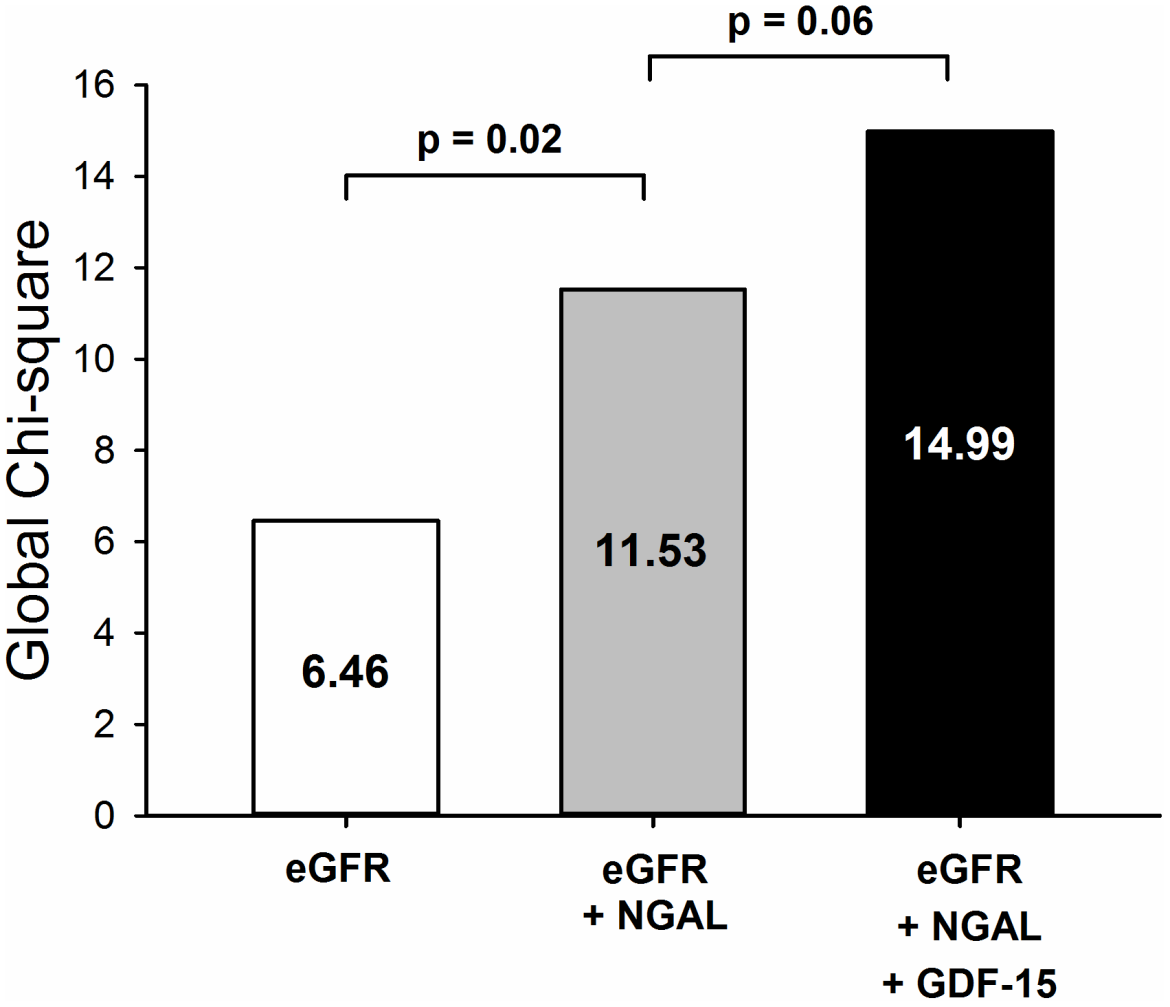
Incremental value of post-operative GDF-15 to predict AKIN after CABG surgery. Bar graph illustrating the change in global χ2 value by the addition of post-operative GDF-15 and/or NGAL to post-operative eGFR. All models were significantly associated with AKI (p<0.05). * = p<0.05 vs eGFR model. § =  p = 0.06 vs eGFR + NGAL model.

Cardiac Troponin I (cTnI) was significantly higher (p = 0.008) on arrival at the ICU (Figure 5A) and remained high the following day. Concerning the early stages of kidney damage, plasma NGAL levels increased significantly (p<0.001) after surgery (Figure 5C) and remained high in the ICU. Moreover, the day after surgery, plasma GDF-15 levels correlated positively and significantly with cTnI and NGAL ICU levels (Figure 5B, D). Among all of the post-operative biomarkers available, only eGFR, NGAL and GDF-15 measured at ICU arrival were significantly associated with the onset of AKI. Given the small size of the population, we performed stepwise multivariate regression beginning with eGFR, and we tested the incremental performance of the model when the two other biomarkers were added (Figure 6). The addition of NGAL to eGFR significantly improved the model’s performance (p = 0.02), as did the addition of GDF-15 levels to eGFR (p = 0.03). When GDF-15 levels were added to the combined model of eGFR+NGAL, there was a trend towards an improvement in the global Chi-square coefficient of the model (p = 0.06).

## Discussion

During CABG, ischemia-reperfusion injury may occur because of elevated intracellular levels of reactive oxygen species and calcium. CPB is associated with an inflammatory response that can generate systemic oxidative stress, which, in turn, causes further damage not only to the heart, but also to other organs. Therefore, it is necessary to identify new biomarkers of inflammation and oxidative stress during this surgical procedure, in order to predict the severity of damage to vital organs.

### Determinants of GDF-15 at baseline

Our data showed that pre-operative GDF-15 levels in patients undergoing bypass surgery were positively associated with age, a history of diabetes, chronic renal failure, high NT-proBNP and plasma CRP levels. Clinical studies have shown that GDF-15 concentrations correlated strongly with age in both healthy and unhealthy adults[15]. Ho *et al.* found that GDF-15 concentrations in apparently healthy elderly adults were higher than those in younger adults [22]. More recently, it was reported that these changes could reflect both cardiovascular and renal perturbations, inflammation and other independent pathophysiological processes[23]. The association between elevated GDF-15 levels and diabetes has also been reported. In obese non-diabetic individuals, GDF-15 could predict future insulin resistance and impaired glucose control[24], and in patients free of clinically-overt cardiovascular disease, GDF-15 was positively associated with age, diabetes, hypertension, worse kidney function[22] and NT-proBNP levels[23]. Indeed, several studies have also reported higher plasma GDF-15 levels in patients with cardiovascular pathologies such as CAD [25], ACS[7], [8], [26] or chronic heart failure[9], [27]. Furthermore, in patients with CAD, GDF-15 concentrations correlated with other markers of inflammation[28], and elevated GDF-15 levels are now considered a strong biomarker of risk progression and mortality in cardiovascular disease[29]–[31]. GDF-15 expression is up-regulated in chronic kidney disease and represents a novel independent serum marker of mortality[13].

### Evolution of GDF-15 during CABG

This is the first report of the time course of GDF-15 during cardiac surgery. Our study demonstrated that plasma GDF-15 levels in humans increased during cardiac surgery associated with CPB. Concerning myocardial IR injury during CABG, the increase in plasma GDF-15 levels that we report is in accordance with experimental findings[6]. Rat cardiomyocytes subjected to simulated IR secreted GDF-15, but only after 3 hours of IR. In an *in vivo* mouse model of cardiac IR injury, myocardial GDF-15 mRNA levels rapidly increased in the ischemic area after both permanent and transient coronary artery ligation. In our study, the median duration of ischemia (cross-clamp time) was 61.7±3.7 minutes, and we examined the levels of GDF-15 after unclamping and after 1 to 2 hours of reperfusion; showing that, in humans, plasma GDF-15 levels increased significantly after a period of myocardial IR.

Heart surgery with CPB is associated with an acute inflammatory response, which has implications for postoperative recovery and myocardial function[32]. Recent findings also suggested that the secreted protein FSTL-1 could be an upstream inducer of GDF15 production and an independent prognostic biomarker in acute coronary syndromes[11]. FSTL1 is a cardiokine, whose cardiac expression levels are upregulated in hypertrophic hearts of mice or in myocardial infarction models[33]. FSTL1 can serve as a useful biomarker of cardiovascular disease given that elevated circulating levels of FSTL1 are found in patients with ACS and associated with chronic HF[34]. In our study performed in patients undergoing bypass surgery, although IND plasma levels of FSTL1 were significantly associated with LVEF (r = −0.385, p =  0.025) and with NT-proBNP concentrations (r = 0.350, p = 0.043), we were unable to demonstrate an association between GDF-15 levels and FSTL-1, at any time during the surgical procedure or the recovery period.

To summarize, our data emphasize the role of GDF-15 in inflammatory situations, and, as suggested by the rapid increase in levels of GDF-15, showed that this induction occurs quickly in cardiac surgery associated with CPB.

### CABG and oxidative stress

Increases in various markers of systemic oxidative stress and in inflammatory products have been shown to occur during CPB[3]. Neutrophils are thought to be the primary source of systemic radical oxygen species (ROS) during heart surgery on CPB [3]. MPO is one of the major neutrophil effector proteins and levels of MPO have been associated with a variety of clinical conditions including systemic inflammation, risk of cardiovascular events, vascular endothelial dysfunction and oxidative stress[35]_ENREF_63. MPO produces hypochlorous acid from hydrogen peroxide and chloride anion during the neutrophil's respiratory burst. In the present work, circulating hydroperoxides did not increase throughout the surgical procedure, even though the plasma antioxidant status significantly decreased and plasma MPO levels increased significantly just after aortic cross clamping. In a previous study, we observed that the PAS decreased during CPB[3], reflecting the reduced capacity of plasma to protect its environment from free-radical aggression; this phenomenon is probably triggered to a large extent by hemodilution. Additionally, it should be noted that mannitol, a well-known non-specific antioxidant, administered just before myocardial reperfusion, could have influenced the level of oxidative stress in these patients. Kempf *et al.* [6] reported that nitro-oxidative stress induced by peroxynitrite was able to induce the expression of GDF-15 in cultured rat neonatal cardiomyocytes. However, at this point in our study, it seems difficult to draw any conclusions on the possible interaction between circulating markers of oxidative stress and GDF-15 levels in the clinical setting of cardiac surgery associated with CPB.

### GDF-15 and markers of renal and cardiac injury during CABG

We did find a close association between high GDF-15 levels and worse eGFR. As our population was small, few adverse events were recorded. There were only 9 AKI events, and the results of the multivariate analysis therefore need to be interpreted with caution. In particular, we were unable to show that the prognostic value of the early measurement of GDF-15 was better than a model that associated two powerful biomarkers of the AKIN. Nonetheless, this result supports our findings that GDF-15 is closely associated with renal function and, in combination with known biomarkers, such as NGAL and eGFR, could be of use for the early detection of AKI. This is in agreement with previous results showing that GDF-15 was a novel independent serum marker of mortality in chronic kidney disease[13]. Correlations between GDF-15 levels and renal function biomarkers, such as creatinine, urea or NGAL levels, became significant during the post-surgery period and the following day, suggesting that GDF-15 possibly originates in the kidney or is influenced by renal function. Elevated urinary levels of GDF-15 were found in type-2 diabetic patients with subnormal eGFR, and were associated with proximal tubule injury[36]. NGAL was originally identified in neutrophil granules but is also expressed in the kidney and liver, and its synthesis is induced in response to inflammation, infection, ischemia, and acute kidney injury[18]. Several studies investigating cardiac surgery patients have shown that NGAL levels in both the blood and urine could be a useful predictive marker of acute kidney injury[19].

While there was no correlation between preoperative GDF-15 and cTnI and CK, we found a significant positive association between GDF-15 and NT-proBNP during the post-operative reperfusion period, and there was a significant positive correlation between ICU GDF-15 and cTnI. In experimental conditions[6], IR injury was shown to induce GDF-15 in cardiomyocytes and mouse hearts. Several clinical studies have clearly demonstrated that GDF-15 was a strong biomarker of cardiovascular disease, and a prognosis marker of fatal events in patients with myocardial infarction[7], [8], [30] and chronic HF[9], [15]. Indeed, plasma GDF-15 is elevated in patients with CAD, and levels are even higher in patients with STEMI. GDF-15 is independently associated with NT-proBNP and cardiac troponin T (cTnT) levels at presentation in STEMI patients[8], [26]. Persisting elevated GDF-15 levels may be related to myocardial injury, revealed by the increase in circulating levels of cTnI. However, to our knowledge, this is the first study to report that elevated GDF-15 levels are positively associated with markers of cardiac injury after CABG with CPB.

### GDF-15 and other risk factors during CABG

We found a significant association between GDF-15 and hemoglobin levels during the post-operative period. GDF-15 is known to be implicated in dyserythropoietic syndromes such as thalassemia or sickle cell disease[37]. Theurl *et al.* demonstrated that GDF15 was significantly increased in chronic anemia in subjects with or without true iron deficiency[38]_ENREF_69. GDF-15 is probably involved in mechanisms that regulate hemoglobin levels; however, further studies are needed to elucidate these pathways.

Finally, our results showed that higher pre-, per- and post-operative GDF-15 levels were significantly associated with higher EuroSCORE values. Our results are in agreement with a recent study showing that pre-operative plasma GDF-15 levels improved the predictive capacity of the additive EuroSCORE. Collectively, these results suggest that the addition of GDF-15 to conventional risk stratification tools could provide important prognostic information[12].

## Conclusions

In conclusion, our prospective study demonstrated for the first time the kinetic increase in plasma GDF-15 levels during cardiac surgery in patients undergoing CABG associated with CPB. We confirm here the important role of GDF-15 as a marker of renal and cardiac injury in patients with CAD who underwent CABG associated with CPB. Given the value of GDF-15 as a marker of heart disease and cardiovascular risk and the prevalence of major adverse cardiovascular events associated with the post-CPB period, it seems important to measure GDF-15 levels in patients undergoing CABG with CPB so as to improve diagnosis and follow-up.
